# Trial of labor after two cesarean sections: A retrospective case–control study

**DOI:** 10.1111/jog.15351

**Published:** 2022-07-06

**Authors:** Rebecca Horgan, Saif Hossain, Adriana Fulginiti, Ariana Patras, Robert Massaro, Alfred Z. Abuhamad, Tetsuya Kawakita, Robert Graebe

**Affiliations:** ^1^ Department of Obstetrics & Gynecology Monmouth Medical Center Long Branch New Jersey USA; ^2^ Department of Maternal Fetal Medicine Eastern Virginia Medical School Norfolk Virginia USA

**Keywords:** cesarean section, labor, trial, vaginal birth after cesarean

## Abstract

**Aim:**

The objective of this study was to compare neonatal and maternal outcomes among women with two previous cesarean deliveries who undergo trial of labor after two cesarean section (TOLA2C) versus elective repeat cesarean delivery (ERCD). Our primary outcome was neonatal intensive care unit (NICU) admission. Secondary outcomes included APGAR score <7 at 5 min, TOLA2C success rate, uterine rupture, postpartum hemorrhage, maternal blood transfusion, maternal bowel and bladder injury, immediate postpartum infection, and maternal mortality.

**Methods:**

This retrospective cohort study was undertaken at a community medical center from January 1, 2008 to December 31, 2018. Inclusion criteria were women with a vertex singleton gestation at term and a history of two prior cesarean sections. Exclusion criteria included a previous successful TOLA2C, prior classical uterine incision or abdominal myomectomy, placenta previa or invasive placentation, multiple gestation, nonvertex presentation, history of uterine rupture or known fetal anomaly. Maternal and neonatal outcomes were assessed using Fisher exact test and Wilcoxon rank sum test.

**Results:**

A total of 793 patients fulfilled study criteria. There were no differences in neonatal intensive care unit admissions or 5‐min APGAR scores <7 between the two groups. Sixty‐eight percent of women who underwent TOLAC (*N* = 82) had a successful vaginal delivery. The uterine rupture rate was 1.16% (*N* = 1) in the TOLA2C group with no case of uterine rupture in the ERCD group. No difference in maternal morbidity was noted between the two groups. No maternal or neonatal mortalities occurred in either group.

**Conclusions:**

There was no difference in maternal or neonatal morbidity among patients in our study population with two previous cesarean sections who opted for TOLA2C versus ERCD.

## Introduction

Cesarean delivery rates are increasing worldwide and are associated with adverse maternal and neonatal outcomes.^1^, ^2^, ^3^, ^4^, ^5^ In the United States, cesarean delivery is the most frequently performed major surgical procedure.^6^ The rate of cesarean delivery increased from 1996 to 2009 where it peaked at 32.9% and has remained relatively stable since then, with a rate of 31.9% in 2018.^7^ Over 90% of women with a previous cesarean section in the United States deliver by repeat cesarean section.^8^ An increasing number of cesarean sections is associated with both maternal and neonatal morbidity.^9^, ^10^, ^11^, ^12^, ^13^, ^14^ A paucity of data exists on women in the United States who attempt trial of labor after two previous cesarean sections (TOLA2C).

An objective of the US Department of Health and Human Resources “Healthy People 2020” was to reduce the rate of cesarean delivery among low‐risk women with a prior cesarean birth by 10%.^15^ Promotion of trial of labor after cesarean section (TOLAC) is one method to potentially reduce the overall cesarean delivery rate.^16^ The American College of Obstetricians & Gynecologists (ACOG) support offering appropriately counseled women with two prior cesarean deliveries TOLA2C which is in agreement with other national guidelines.^17^ However, most women with two prior cesarean deliveries are not offered TOLA2C. This may be due to provider concern regarding risks such as uterine rupture and insufficient provider knowledge regarding the risks and benefits of TOLAC with two prior cesarean sections, compared to elective repeat cesarean delivery (ERCD).^18^ Successful vaginal birth after two prior cesarean sections has the potential to theoretically decrease both the overall rate of cesarean sections and the complications associated with multiple cesarean sections.^18^

Previous literature comparing TOLA2C and ERCD is limited, in particular among US populations. Some authors have previously reported increased maternal morbidity associated with TOLA2C versus ERCD whereas others have not.^17^, ^18^, ^19^ The objective of this study is to examine maternal and neonatal outcomes among women with two previous cesarean deliveries who underwent TOLA2C versus ERCD at our institution. Our primary outcome was neonatal intensive care unit (NICU) admission. Secondary outcomes included APGAR score <7 at 5 min, TOLA2C success rate, uterine rupture, postpartum hemorrhage, maternal blood transfusion, maternal bowel and bladder injury, immediate postpartum infection, maternal mortality.

## Methods

A retrospective case control study was undertaken at Monmouth Medical Center in New Jersey from January 1, 2008 to December 31, 2018 inclusive. Monmouth Medical Center is a tertiary community teaching hospital with approximately 5500 deliveries per annum. All women admitted to labor and delivery with a history of a prior cesarean delivery during the study period were identified using the International Classification of Disease codes 9 and 10; 654.21, O34.211, and O34.212 and electronic medical records were reviewed to identify all women with a history of two prior cesarean deliveries. Inclusion criteria were women with a cephalic singleton gestation at term and a history of two prior cesarean sections. Term gestation was defined as 37 weeks or greater gestational age. Exclusion criteria included a previous successful TOLA2C, prior classical uterine incision or abdominal myomectomy, fetal malpresentation, placenta previa or suspected invasive placentation, multiple gestation, history of uterine rupture or known fetal anomaly. Uterine rupture was strictly defined as full‐thickness separation of the prior uterine scar found at the time of surgery and clinically correlating evidence of uterine rupture.

Women who attempted a trial of labor after two prior cesarean deliveries and who fulfilled our inclusion criteria were further categorized as either successful or failed TOLA2C. Women who opted to undergo an ERCD and fulfilled our inclusion criteria were placed in the second group. Women opting for an ERCD were defined as patients who did not wish to undergo a trial of labor and also included women who presented in labor but declined TOLA2C.

Demographic data for each patient was collected including age, race, body mass index (BMI) on admission to labor and delivery, gravida, parity. For each patient, the indication for the two prior cesarean sections was recorded, history of previous vaginal delivery and history of Vaginal birth after cesarean delivery (VBAC). For patients who opted to undergo a TOLA2C, patients were further categorized as spontaneous labor or induction of labor. Maternal complications including postpartum hemorrhage, blood transfusion, infection during hospital stay, bowel injury, bladder injury, and hysterectomy were collected. We defined postpartum hemorrhage as a cumulative blood loss greater than 1000 mL. Neonatal electronic medical records of all women included in the study were reviewed to identify Appearance, Pulse, Grimace, Activity, Respiration (APGAR) scores, birthweight, perinatal deaths, and neonatal intensive care unit (NICU) admissions. Electronic medical record review and data extraction were performed by four resident physicians. Maternal and neonatal outcomes were assessed using Fisher exact test and Wilcoxon rank sum test. Statistical significance was defined as *p* < 0.05. Our study was approved by the Institutional Review Board at Monmouth Medical Center.

## Results

**FIGURE 1:**
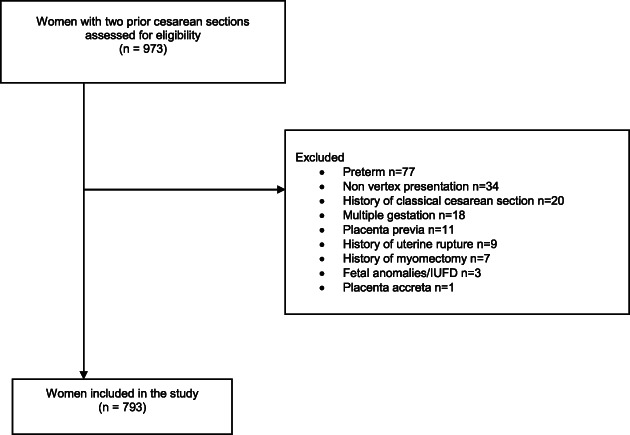
Women with two prior cesarean sections assessed for study eligibility

A total of 6788 patients who were admitted to labor and delivery during the study period had a history of a prior cesarean delivery. Nine hundred and seventy‐three women had a history of two prior cesarean deliveries. One hundred and eighty women with two prior cesarean deliveries did not fulfill our inclusion criteria and were thus excluded (Figure 1). Seven hundred and ninety‐three women had a history of two prior cesarean sections and fulfilled our inclusion criteria. Eighty‐two women (10.3%) with two prior cesarean deliveries opted to undergo TOLA2C and 711 women (89.7%) opted for ERCD.

There was no difference in age, race, or BMI noted between the TOLA2C and ERCD groups but women who opted to undergo TOLA2C were more likely to have had a prior vaginal delivery and a prior VBAC (Table 1). There was no difference in our primary outcome of NICU admissions (5.9% vs. 4.9%, *p* = 0.99) in the ERCD versus TOLA2C group (Table 2). There was no difference in the rate of APGAR scores <7 at 5 min between the two groups and no neonatal deaths occurred in either group (Table 2).

**TABLE 1:** Maternal demographics

	ERCD, *n* = 711	TOLA2C, *n* = 82	*p*‐Value
Mean age (SD)	33.0 (4.8)	33.1 (4.7)	0.71
Race, *n* (%)			0.14
Asian	9 (1.3)	0 (0.0)	
Black	58 (8.2)	2 (2.4)	
Hispanic	122 (17.2)	10 (12.2)	
Other	37 (5.2)	3 (3.7)	
White	483 (68.1)	67 (81.7)	
Mean BMI (SD)	32.0 (7.1)	32.0 (5.6)	0.53
Mean parity (SD)	2.1 (0.6)	4.1 (2.8)	<0.001
Prior vaginal delivery, *n* (%)	46 (6.6)	50 (61.0)	<0.001
Prior history of VBAC, *n* (%)	10 (1.4)	46 (56.1)	<0.001

Abbreviations: BMI, body mass index; ERCD, elective repeat cesarean delivery; TOLA2C, trial of labor after two prior cesarean sections; VBAC, vaginal birth after cesarean.

**TABLE 2:** Maternal and neonatal outcomes

	ERCD, *n* = 711	TOLA2C, *n* = 82	*p*‐Value
APGARS @ 5 min <7, *n* (%)	1 (0.1)	0 (0.0)	0.99
NICU admission, *n* (%)	42 (5.9)	4 (4.9)	0.99
Neonatal death, *n* (%)	0 (0)	0 (0)	n/a
Mean birthweight (SD)	3434.1 (447.4)	3455.8 (416.6)	0.68
PPH, *n* (%)	52 (7.3)	3 (3.7)	0.26
Blood transfusion, *n* (%)	15 (2.1)	2 (2.4)	0.69
Bowel injury, *n* (%)	1 (0.1)	0 (0.0)	0.99
Bladder injury, *n* (%)	2 (0.3)	1 (1.2)	0.28
Infection, *n* (%)	6 (0.8)	0 (0.0)	0.99
Hysterectomy, *n* (%)	1 (0.1)	0 (0.0)	0.99
Uterine rupture, *n* (%)	0 (0)	1 (1.16)	0.99

Abbreviations: ERCD, elective repeat cesarean delivery; PPH, postpartum hemorrhage; TOLA2C, trial of labor after two prior cesarean sections.

About 69.5% of women who underwent TOLA2C (*N* = 82) had a successful vaginal delivery. The indications for a repeat cesarean section in the TOLA2C group were arrest of dilation (*n* = 12), nonreassuring fetal heart tracing (*n* = 11), arrest of descent (*n* = 1), uterine rupture (*n* = 1). The uterine rupture rate was 1.2% (*n* = 1) in the TOLA2C group (Table 2). No cases of uterine rupture occurred in the ERCD group but there was one unplanned hysterectomy (Table 2).

The indications for the first and second cesarean section for both groups are listed in Table 3. No difference in maternal blood transfusions, postpartum hemorrhage, immediate postpartum infection, peripartum hysterectomy, bladder injury, or bowel injury was noted between the ERCD and TOLA2C groups (Table 2).

**TABLE 3:** Indication for first and second cesarean delivery

	First cesarean delivery		Second cesarean delivery	
	ERCD, *n* = 711	TOLA2C, *n* = 82	ERCD, *n* = 711	TOLA2C, *n* = 82
NRFHT	118 (16.6)	15 (18.3)	19 (2.7)	10 (12.2)
Arrest of dilation	139 (19.5)	9 (11.0)	37 (5.2)	9 (11)
Breech	59 (8.3)	13 (15.9)	15 (2.1)	8 (9.8)
Arrest of descent	44 (6.2)	3 (3.7)	4 (0.6)	1 (1.2)
LGA	23 (3.2)	4 (4.9)	2 (0.3)	0 (0)
Placental abruption	12 (1.7)	1 (1.2)	9 (1.3)	1 (1.2)
Elective	12 (1.7)	1 (1.2)	472 (66.4)	12 (14.6)
Placenta previa	5 (0.7)	0 (0)	4 (0.6)	1 (1.2)
Other	19 (2.6)	3 (3.6)	4 (0.6)	1 (1.2)
Unknown	280 (39.4)	33 (40.2)	145 (20.4)	33 (40.2)
Total	711 (100)	82 (100)	711 (100)	82 (100)

Abbreviations: ERCD, elective repeat cesarean delivery; LGA, large for gestational age; NRFHT, nonreassuring fetal heart tracing; TOLA2C, trial of labor after two prior cesarean sections.

Women who underwent successful TOLA2C had a lower mean BMI on admission to labor and delivery (31.0 vs. 34.5, *p* = 0.04), lower mean neonatal birth weight (3351 vs. 3681 g, *p* = 0.01) and were more likely to have had a previous vaginal delivery and a previous successful VBAC in comparison to women who had an unsuccessful TOLA2C (Table 4). Induction of labor or augmentation of labor was associated with a greater likelihood of failed TOLA2C (Table 4).

**TABLE 4:** Failed TOLA2C versus successful TOLA2C

	Failed TOLA2C, *N* = 25 [*n* (%)]	Successful TOLA2C, *N* = 57 [*n* (%)]	*p*‐Value
Mean age (SD)	32.8 (5.1)	33.2 (4.6)	0.95
Race, *n* (%)			0.06
Asian	0 (0.0)	0 (0.0)	
Black	2 (8.0)	0 (0.0)	
Hispanic	5 (20.0)	5 (8.8)	
Other	1 (4.0)	2 (3.5)	
White	17 (68.0)	50 (87.7)	
Mean BMI (SD)	34.5 (6.3)	31.0 (4.9)	0.04
Prior vaginal delivery, *n* (%)	7 (28.0)	43 (75.4)	< 0.001
Prior VBAC, *n* (%)	5 (20.0)	41 (71.9)	< 0.001
Induction of labor, *n* (%)	7 (28.0)	4 (7.0)	0.03
Augmented labor, *n* (%)	14 (56.0)	15 (26.3)	0.001
Mean birthweight (SD)	3681.3 (445.1)	3350.9 (362.1)	0.01
APGAR <7 at 5 min	0 (0.0)	0 (0.0)	n/a
NICU admission	2 (8.0)	2 (3.5)	0.58
Perinatal death	0 (0.0)	0 (0.0)	n/a
PPH	3 (12.0)	0 (0.0)	0.03
Blood transfusion, *n* (%)	2 (8.0)	0 (0.0)	0.09
Bladder injury, *n* (%)	1 (4.0)	0 (0.0)	0.99
Bowel injury	0 (0.0)	0 (0.0)	n/a
Infection	0 (0.0)	0 (0.0)	n/a
Peripartum hysterectomy	0 (0.0)	0 (0.0)	n/a

Abbreviations: APGAR, appearance, pulse, grimace, activity, respiration; BMI, body mass index; NICU, neonatal intensive care unit; PPH, postpartum hemorrhage; VBAC, vaginal birth after cesarean; TOLA2C, trial of labor after two prior cesarean sections.

## Discussion

Our study demonstrates that for women with a history of two previous cesarean deliveries, TOLA2C and ERCD have comparable maternal and neonatal outcomes. Our TOLA2C success rate of 69.5% is consistent with a 2010 meta‐analysis by Tahseen et al. which noted a success rate of 71.1% but significantly higher than the 53.6% success rate cited in a recent US population‐based cohort study.^18^, ^20^ The uterine rupture rate among our study population was 1.2%. Previous literature has demonstrated that uterine rupture poses the greatest risk of adverse neonatal outcome with an overall perinatal mortality rate of 6%.^10^ Tahseen et al. found a greater than twofold risk of uterine rupture among women with more than one prior cesarean section (1.59% vs. 0.72%).^18^ In contrast, this finding was not replicated by the Maternal‐Fetal Medicine Units Network (MFMU) cesarean registry which found no increased risk of rupture among women with one versus multiple cesarean sections.^21^

Previous data examining maternal morbidity associated with TOLA2C versus ERCD has been conflicted with some studies demonstrating increased composite maternal morbidity among patients who opted for ERCD.^22^ However, these findings were not sustained by larger population‐based cohorts or meta‐analyses.^18^, ^23^ Similarly, we noted no difference in maternal outcomes between the TOLA2C and ERCD groups. The neonatal outcome after TOLA2C or ERCD were also comparable among our patient population and this is consistent with previous data.^18^ Previous vaginal delivery increased the likelihood of successful TOLA2C among our patient population whereas induction of labor after two prior cesarean sections adversely affected successful TOLA2C. Previous studies examining trial of labor after one cesarean section have found induction of labor was associated with decreased a TOLAC success rate.^21^

The indication for the second cesarean section was elective choice in 66% in the ERCD group versus 15% in TOLA2C group (Table 3). This illustrates the importance of offering women TOLAC after one prior cesarean section in an attempt to decrease the risks associated with higher order cesarean sections. Our study also highlights the importance of offering attempts at vaginal delivery as an option to women with two prior cesarean sections, particularly if she has had a previous vaginal delivery and spontaneous labor occurs. The American College of Obstetricians and Gynecologist's states that it is “reasonable to consider” TOLA2C which is consistent with guidelines from both the Royal College of Obstetricians & Gynecologists in the United Kingdom and the Society of Obstetricians and Gynecology of Canada.^1^, ^24^ Previous literature has demonstrated that outcomes of trial of labor after cesarean section are similar regardless of whether a woman has had one or two prior cesarean sections.^23^ The MFMU prediction model has also been shown to be predictive for those with either one or two prior cesarean sections.^25^ However, recent literature has questioned the validity of VBAC prediction calculators, particularly among patients for whom the predicted VBAC success rate is low.^26^, ^27^

The biggest strength of this study is the lack of confounding effect for age, race, and BMI between the two study groups and the large study population size. A limitation of this study includes incomplete medical records before the generalized use of electronic medical records. Thus, indications for prior cesarean sections were often not reported. Another limitation is that the researchers of this study were not present during the time the option of repeat cesarean section or TOLA2C were discussed. Therefore, it remains unknown if all eligible women were offered TOLA2C, potentially causing selection bias. Finally, as the patients who deliver at our hospital attend a variety of different obstetric practices for their postpartum care, we did not have access to their medical records to follow up outcomes beyond the immediate postpartum period. Thus, complications such as postpartum infection rates may be underestimated.

In conclusion, there was no difference in maternal or neonatal morbidity among patients in our study population with two previous cesarean sections who opted for TOLA2C versus ERCD. Increasing the rate of TOLA2C has the potential to decrease overall cesarean section rates and thus the morbidity associated with increasing cesarean sections. TOLA2C should be discussed as an option with women, where appropriate.

## Author contributions

Dr Horgan was involved in study design, data collection, analysis of data and drafting of the manuscript. Dr Hossain was involed in study design and data collection. Dr's Fulginiti and Patras were involved in data collection. Dr Massaro was involved in study design, revision of the manuscript and supervision. Dr Abuahamad was involved in revision of the manuscript. Dr Kawakita was involved in drafting of the manuscript and revision of the manuscript. Dr Graebe was involved in study design, revision of the manuscipt and supervision.

## Conflict of interest

No potential conflict of interest was reported by the authors.

## Data Availability

Data available on request from the authors.
